# Progress in Colloid Delivery Systems for Protection and Delivery of Phenolic Bioactive Compounds: Two Study Cases—Hydroxytyrosol and Curcumin

**DOI:** 10.3390/molecules27030921

**Published:** 2022-01-29

**Authors:** Francesca Cuomo, Silvio Iacovino, Pasquale Sacco, Antonella De Leonardis, Andrea Ceglie, Francesco Lopez

**Affiliations:** 1 Department of Agricultural, Environmental and Food Sciences (DiAAA) and Center for Colloid and Surface Science (CSGI), University of Molise, Via De Sanctis, 86100 Campobasso, Italy; francesca.cuomo@unimol.it (F.C.); s.iacovino@studenti.unimol.it (S.I.); antomac@unimol.it (A.D.L.); 2 Department of Life Sciences, University of Trieste, Via Licio Giorgieri 5, 34127 Trieste, Italy; psacco@units.it; 3 Department of Chemistry “Ugo Schiff”, Center for Colloid and Surface Science (CSGI), University of Florence, Via della Lastruccia 3, 50019 Sesto Fiorentino, Italy; ceglie@csgi.unifi.it

**Keywords:** colloids, phenolic compounds, drug delivery, hydroxytyrosol, curcumin

## Abstract

Insufficient intake of beneficial food components into the human body is a major issue for many people. Among the strategies proposed to overcome this complication, colloid systems have been proven to offer successful solutions in many cases. The scientific community agrees that the production of colloid delivery systems is a good way to adequately protect and deliver nutritional components. In this review, we present the recent advances on bioactive phenolic compounds delivery mediated by colloid systems. As we are aware that this field is constantly evolving, we have focused our attention on the progress made in recent years in this specific field. To achieve this goal, structural and dynamic aspects of different colloid delivery systems, and the various interactions with two bioactive constituents, are presented and discussed. The choice of the appropriate delivery system for a given molecule depends on whether the drug is incorporated in an aqueous or hydrophobic environment. With this in mind, the aim of this evaluation was focused on two case studies, one representative of hydrophobic phenolic compounds and the other of hydrophilic ones. In particular, hydroxytyrosol was selected as a bioactive phenol with a hydrophilic character, while curcumin was selected as typical representative hydrophobic molecules.

## 1. Introduction

Modern trends in food science respond to the growing demand of consumers for a healthier lifestyle, associated with the consumption of healthier foods [1]. In this context, a great deal of attention has been paid to the development of food-grade colloid systems that can protect and deliver functional compounds, and which, at the same time, can be used as suitable nutraceutical carriers [2]. The extent to which applications of colloid-mediated delivery systems can be actively incorporated into practical food technology is constantly evolving and remains a fascinating task. Nutraceutical compounds can act as antioxidant and anti-inflammatory agents that are able to extend the shelf life of food formulations and to modulate various human diseases [3,4,5,6]. Thanks to these effects, such bioactive molecules represent a benefit to human health; however, to be effective, adequate formulations are required to improve their solubility, chemical stability, and bioavailability. These important parameters are needed to preserve their bioactivity to thus be available to consumers.

Food colloids are made of dispersions of at least two immiscible phases, such as solid particles in a liquid matrix, lipophilic phases emulsified in an aqueous continuous phase, gas bubbles entrapped in solid or liquid matrices, etc. These systems find applications in many food products [7]. By mixing biopolymers, for example, water and oil, emulsions can be made. Emulsions are commonly used in many chemical and pharmaceutical industries, in food chemistry, as well as in biotechnological applications [8,9,10,11]. An important issue related to emulsions is their instability to environmental stresses and their effects on physico-chemical properties.

To improve the stability of food colloids, surface-active agents are used [12,13]. Among these, the molecules used in foods are proteins, polysaccharides, and low molecular weight surfactants. The common features of all these compounds is the coexistence of hydrophilic and hydrophobic parts in their structures [14]. The importance of surface-active agents in the manufacture of foods, such as sauces, creams, candies, and packaged processed foods, has been known for almost a century [15,16].

In addition, other molecules of polymeric nature can be used, and have various functions, such as gelling, thickening, serving as stabilizing agents, they can be added to modify the rheology of aqueous suspensions, improve texture properties, slow down starch degradation, and increase moisture retention [17]. The major source of polymers for food colloids comes from plants and gums, and are derived from exudates thereof [18].

Encapsulation is a technique used to protect host molecules, slowing down or preventing degradation processes under adverse conditions in food products, or in the upper gastrointestinal tract. Host molecules can be hydrophilic or hydrophobic, such as vitamins, peptides, antioxidants, or probiotics. In a complex system, hydrophilic molecules are encapsulated in the aqueous part, and lipophilic ones in the oil phase. Examples of complex structures are emulsions and multilayer emulsions [19,20]. Several researchers have shown that delivery systems can improve the handling, solubility, chemical stability, and bioavailability of various lipophilic and hydrophilic bioactive compounds, such as polyphenols [21].

Among the water-soluble bioactive compounds, an important molecule found in nature is hydroxytyrosol (HYT) [22]. Hydroxytyrosol (3,4-dihydroxyphenylethanol, 3,4-DHPEA) is a phenol compound mainly found in components and products of olive plant (Olea europaea), especially in olive fruits, leaves and extra virgin oil. Generally, HYT is found as a constituent of complex molecules (HYT derivatives), such as oleuropein and other secoiridoids [23,24,25,26,27,28]. HYT in free and derivative form is characterized by strong antioxidant properties [29], indeed, the European Food Safety Authority (EFSA) states that a daily intake of 5 mg of HYT and its derivatives are able to prevent low density lipoprotein (LDL) oxidation [15].

Curcumin (CUR) is a water-insoluble bioactive compound extracted from the rhizome of Curcuma longa [30]. Its use is strongly associated with anti-inflammatory, anti-carcinogenic, and antimicrobial properties [31,32]. CUR is recognized to have a high bioactivity among the curcuminoids and a low oral toxicity [33]. It is also considered as effective against colorectal [34] and pancreatic cancers [35] due to its capacity to meddle with different biochemical pathways. As a consequence, considering the health benefits [36], there is increasing interest in using CUR as a bioactive agent in functional foods [37]. Nevertheless, due the low water solubility of CUR hard to incorporate into foods and beverages, while its low oral bioavailability may reduce its biological activity [33,38,39]. The key aspects that limit the CUR bioavailability are the low solubility in gastrointestinal fluids and its propensity to experience chemical transformations within the gastrointestinal tract [40]. These issues may be addressed by encapsulating CUR in food-grade delivery systems, such as liposomes, nano-complexes, colloidosomes [41,42], emulsions [20,43], nanoemulsions [44,45], or biopolymer nanoparticles [46,47].

The aim of this review is to present the recent advances in the field of protection and delivery of bioactive compounds through colloid systems. In particular, emulsions, particles, and liposomes used as delivery systems are discussed with particular attention to the biomolecules hydroxytyrosol and curcumin.

## 2. Colloid-Mediated Delivery Systems

**Scheme 1:**
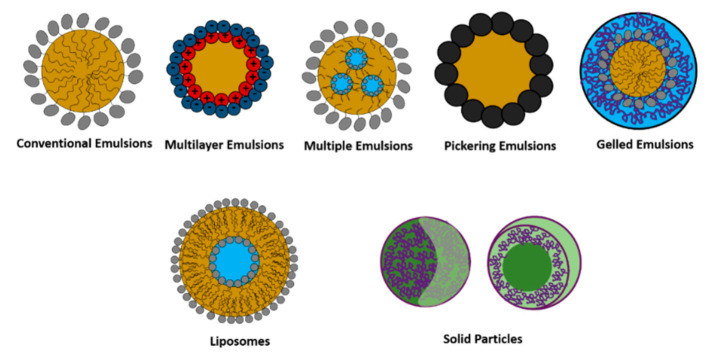
Main colloid-mediated delivery systems.

Various colloid-mediated formulations have been proposed for the vehiculation of hydrophobic and hydrophilic bioactive compounds [48]. Scheme 1 shows an illustration representing the systems analyzed in this review.

Among food-grade emulsifiers lecithin and the mono- and di-glycerides of fatty acids, medium-chain triglycerides (MCT), isopropyl myristate (IPM) for oils, ethanol (in low concentrations), and glycerol for aqueous phases are some of the materials approved by the EFSA and are generally recognized as safe (GRAS) [49].

Food-grade biopolymers include organic polymers, which are widely used to produce a variety of delivery systems suitable for the encapsulation, protection, and delivery of bioactive substances. Considering that oral delivery systems of dietary supplements should be made from food-grade ingredients using economical and reliable processing methods, one of the most promising approaches to produce food-grade colloid delivery systems is to use biopolymers, such as proteins and polysaccharides, as building blocks [8,10,11,50]. In this case, biopolymer-based delivery systems can be assembled from these “ingredients”, using a variety of bottom-up and top-down methods, including controlled aggregation, segregation, and/or careful disruption of the biopolymers. In the next sections, we provide a brief description of the delivery systems mentioned in this review.

### 2.1. Conventional Emulsions

Emulsions are constituted by three main components [51]: oil (*O*), emulsifier, and water (*W)*. Depending on their reciprocal ratios, these organizations can be made by continuous dispersions of water droplets in oil (*W*/*O*), or oil droplets dispersed in water (*O*/*W*), or by bicontinuous systems in the case that they contain almost equal amounts of oil and water [52]. The principal and simpler types of emulsions are macroemulsions, microemulsions, and nanoemulsions.

Macroemulsions are characterized by a mean dimension of the dispersed phase on the order of μm, while in microemulsions the dispersed phase is about 2–50 nm [53], and in nanoemulsions it is about 50–300 nm. Hydrophilic compounds can be dissolved in the aqueous phase, while hydrophobic compounds can be dissolved in the oil phase. Nowadays, there is growing interest in the formulation of food-grade emulsions as reservoirs and carriers of bioactive compounds, including phenolic antioxidants.

### 2.2. Multilayer Emulsions

Multilayer emulsions are systems in which droplets are surrounded by two or more layers, generally produced by a layer-by-layer technique [54]. This approach consists of two or more steps of layer adsorption, where charged emulsifiers are first applied to the surface of the droplets, and then charged emulsifiers or polymers are attracted to the previously adsorbed layer. The formation of multilayer interfaces is mainly due to the electrostatic forces triggered by pH and ionic strength. Variations in these parameters are able to modify the structure of the interfacial layer (charge density, thickness, and compactness) and the interaction between the emulsion constituents. It has been reported that emulsions containing oil droplets surrounded by a multilayer interface have better stability against external factors, such as heating or thawing, and are more effective in protecting nutrients from degradation. Therefore, multi-layered emulsions can respond differently to environmental factors, allowing for an improved delivery of the contained active ingredients. A few effective multilayer emulsions as delivery systems have been reported, which are composed of pectin in combination with whey protein isolate [55], lecithin [56], and β-lactoglobulin [57].

### 2.3. Multiple Emulsions

Multiple emulsions (or double emulsions) are more complex liquid dispersions consisting of beads containing small droplets of liquid, which are then re-dispersed in the same liquid (continuous phase). An example of a double emulsion is water-in-oil-in-water (*W*/*O*/*W*) emulsions, where water droplets are dispersed in oil droplets, which are, in turn, dispersed in a continuous aqueous phase. Following the same concept, in oil-in-water-in-oil (*O*/*W*/*O*) emulsions, the continuous phase is an organic solvent. For the realization of these systems, the inner phase consists of an aqueous (for hydrophilic compounds) or organic solution (for hydrophobic compounds), while the outer phase is prepared by dissolving the surfactants [58].

From a practical point of view, this type of system offers several advantages in the delivery and protection of labile bioactive compounds during storage or digestion [59]. Multiple emulsions in line with the other systems allow for the delivery of both hydrophilic and hydrophobic bioactive compounds in aqueous and organic media, respectively [60].

### 2.4. Pickering Emulsions

Pickering emulsions (PE) are defined as emulsions stabilized by colloid micro- or nano-solid particles at the oil–water interface [61,62]. Overall, the available literature in this field has considered a large number of colloid particles with a range of physicochemical properties, such as size or wettability. In Food industry, various substances are commonly used as stabilizers to produce PE, such as starch particles [63] or chitin nanocrystals [64]. Recently, the effectiveness of using soluble and insoluble whey protein concentrate gum arabic complexes has also been reported [65]. The amount of interface of a PE is mechanically stronger compared with a conventional emulsion and can provide sufficient steric repulsive forces to prevent coalescence of the droplets. It has been reported that this system can better transport bioactive molecules, such as curcumin [66], fatty acids [67], and retinol [68]. It has also been reported that colloid particles involved in stabilization can be used as interfacial reservoirs of bioactive compounds [69]. Ultimately, the use of PE loaded with bioactive compounds lead to a dual functionality, as they provide excellent physical and oxidative stability to emulsions and also serve as reservoirs for bioactive molecules.

### 2.5. Gelled Emulsions

Gelled emulsions are complex materials characterized by the simultaneous presence of emulsion and gel structures [70]. In this type of systems, emulsion droplets are enclosed in a continuous hydrogel matrix that exhibits positive plastic properties. Gelled emulsions exhibit several physical and structural properties, such as stability, viscoelasticity, encapsulation efficiency, minimization of phase separation, better control of release kinetics, and protection of labile components during storage or digestion [71].

### 2.6. Liposomes

Liposomes are nano- to micro-sized vesicles, comprising a phospholipid bilayer that surrounds an aqueous core. In such structures, the core encapsulates water-soluble drugs and the hydrophobic area is responsible for entrapping insoluble agents. Thus, such aggregates are able to include, and eventually deliver, aqueous or lipid molecules. Within the bilayer, the hydrophobic tails of the phospholipid groups face each other, while the hydrophilic heads face the inner core and the outer matrix [72]. This particular structure, allows hydrophilic substances to be incorporated directly into the core, while the hydrophobic substances can be distributed in the bilayer area. Their ability to transport both hydrophilic and lipophilic fractions, the absence of cytotoxicity, and their biocompatibility and biodegradability are the reasons for the frequent use of these soft particles in the field of delivery of bioactive compounds [73]. In addition, the release of encapsulated payloads can be modulated by changing the fluidity of the liposomal membrane, depending on the amount of incorporated cholesterol [45]. For these reasons, liposomes are applied and used in a variety of sectors, such as in the pharmaceutical industry, to increase the bioavailability of delivered molecules [45,72,74].

### 2.7. Solid Particles

Bioactive compounds can also be entrapped within with a polymeric shell or, alternatively, embedded in a polymeric matrix [59]. Generally, we refer to micro-encapsulation for products with a diameter between 1 and 1000 µm; instead, the term nano-encapsulation is used when an aggregate’s diameter is between 10 and 1000 nm. The end products of the encapsulation process are, thus, referred to as micro- or nanoparticles, depending on the scale, and include both spheres and capsules [75]. Micro- and nano-particles have been widely studied for drug delivery and various “ingredients” have been used for their realization [76]; for example, for systems where the bioactive molecule can be enclosed in a cavity (capsule) surrounded by a unique polymer membrane (wall or shell, solid), while spheres are polymeric matrix systems in which an active ingredient is uniformly dispersed [77]. From a general point of view, a capsule has an inner core containing the bioactive compound and an outer solid polymer shell, while a sphere consists of a solid matrix in which the active ingredient is dispersed [45].

In recent years, various encapsulated systems have been proposed for the delivery of hydrophobic and hydrophilic bioactive compounds via encapsulation processes, mainly differing in terms of the polymeric matrix [78]. In general, emulsion-based techniques [79] and nanoprecipitation [80] are the commonly used methods for producing bioactive-loaded particles. Among these, the advantage of using emulsions is that these systems allow encapsulation of both hydrophilic and lipophilic molecules, depending on the choice of dispersed and continuous phases and the hydrophilic or lipophilic properties of the selected bioactive compound. For example, for hydrophobic molecules, the micro- or nano-precipitation methods resulted as being simpler and less expensive, and do not require external energy inputs through homogenization or sonication [81].

## 3. Phenolic Compounds

**Scheme 2:**
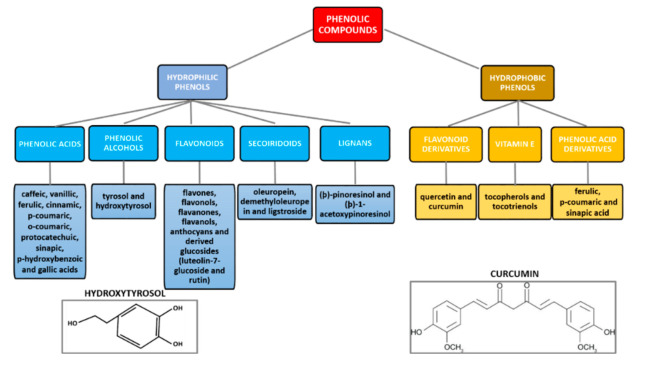
Main phenolic compounds.

Phenolic compounds represent the most important class of secondary metabolites that act as antioxidants at low concentrations [82]. The delivery of these molecules through efficient carriers represents a great opportunity to improve human health. Phenols can be classified based on their origin, biological activity, chemical structure, and affinity to aqueous and oily media (Scheme 2).

In Scheme 2, hydrophilic phenols, such as phenolic acids, phenolic alcohols, flavonoids, secoiridoids, and lignans, are reported [83]. These compounds are secondary aromatic plant metabolites, found in a variety of plants, frequently associated with the color and sensory properties of foods too.

Important phenolic alcohols found in extra virgin olive oils are HYT and tyrosol [84]. These compounds, together with the elenolic acid or its derivatives found in the structure of different secoiridoids like oleuropein, demethyloleuropein, and ligstroside [85]. An example of bioactive secoiridoid is the dialdehydic form of decarboxymethyl elenolic acid linked to hydroxytyrosol or tyrosol. Flavonoids mainly include compounds such as flavones, flavonols, flavanones, flavanols, anthocyans, and derived glucosides (such as luteolin-7- glucoside and rutin) [86]. Lignans are polyphenols encompassing (þ)-pinoresinol and (þ)-1-acetoxypinoresinol [87]. Hydrophobic phenolic compounds (Scheme 2) mainly include flavonoid derivatives, which become more hydrophilic through the glycosylation of hydroxyl groups. Among them, quercetin and curcumin are very important bioactive molecules. Lipophilic phenols also include phenolic acid derivatives of which the hydrophilicity is reduced through the esterification of carboxyl groups, as in the case of ferulic acid, p-coumaric acid, and sinapic acid [88,89].

### 3.1. Hydrophilic Phenols: The Case of Hydroxytyrosol

Presently, various HYT formulations are already on the market as supplements, such as Oleaselect^TM^ (Indena, Milan, Italy) [90], which is based on HYT-rich extracts from solid olive residues, or Micotirosolo (NutraLabs, Modena, Italy), or Hidroxitirosol plus+ (Granatum, Murcia, Spain).

HTY is a stated resource as a dietary supplement, thus, based on the following reported current-delivery systems, innovations for novel supplementation patterns and formulations represent an important nutraceutical molecule to be analyzed. In this respect, various systems were developed for HYT protection and delivery. Table 1 summarizes the main colloid-based delivery systems reported in this review.

#### 3.1.1. Macro and Multiple Emulsions for HYT

Among the available approaches for the delivery of HYT, emulsion-based systems represent one of the most widely studied solutions. Chatzidaki and co-workers [91] developed two edible *W*/*O* dispersions: an emulsion that remains kinetically stable and a microemulsion that is spontaneously formed, transparent, and thermodynamically stable. Both systems contained medium chain triglycerides (MCT) as a continuous phase and were used as carriers of HYT. The results indicated that both systems exhibited good potential for food applications, with the emulsion providing a slightly better HYT release, while the microemulsion provided a better storage ability owing to the higher thermodynamic stability. The work of Flaiz et al. [93] showed that *W*/*O*, *W*/*O*/*W* and gelled *W*/*O*/*W* dispersions can be employed as storage and release system of HYT for food applications. Encapsulated HYT showed a good scavenging activity against free radicals and decreasing permeability due to the interactions of bioactive compounds with the surfactants used in the formulation. In multiple emulsions (*W*/*O*/*W*) for HYT, the delivery and antioxidant ability loss was higher due to the higher compartmentalization degree, compared to conventional *W*/*O* emulsions.

**Figure 1:**
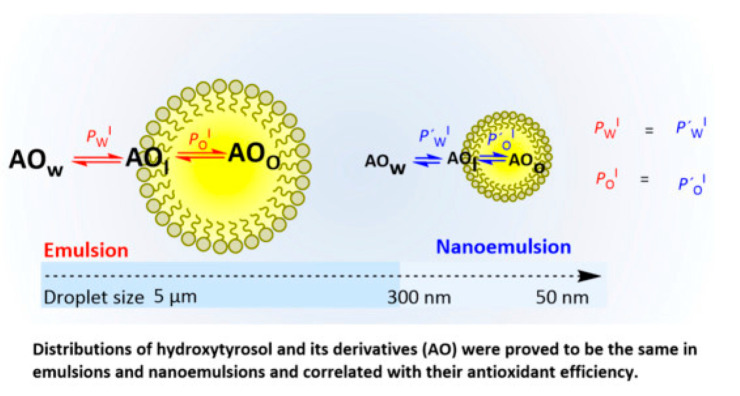
From “Interfacial concentrations of hydroxytyrosol derivatives in fish oil-in-water emulsions and nanoemulsions and its influence on their lipid oxidation: droplet size effects” (reproduced with permission from [92]. Copyright 2020, MDPI).

In a parallel study, Almeida et al. [95] determined the interfacial molarities of the HYT and HYT fatty acid esters, with chain lengths of 1 to 16 carbons, in intact olive *O*/*W*/Tween 20 emulsions. The overall findings highlighted that the distribution and interfacial molarities were consistent with the “cut-off” effect of a pseudo-phase model, and that it was simply a natural consequence of the differential solubility of antioxidants in the aqueous, interfacial and oil regions of the emulsion. Costa et al. [92] employed a set of HYT esters with different hydrophobicity and fish *O*/*W* emulsified systems containing droplets of different sizes to evaluate the effect of droplet size (emulsion vs. nanoemulsion, see Figure 1), surfactant, and oil volume fractions on oxidative stability. The results showed a correlation between antioxidant efficacy and the concentration of antioxidants in the intermediate zone. Interestingly, in both emulsified systems, the highest interfacial concentration and antioxidant efficacy was found for HYT octanoate.

In addition, multiple emulsions have been proposed by Cofrades et al. [94] to evaluate the oxidative stability of lipid-cooked meat systems, in which pork back fat was replaced by a double emulsion prepared with HYT within an inner aqueous phase and chia oil as the lipid phase. The reported results demonstrated that multiple emulsions prepared with chia oil had good stability and a homogeneous structure. Strikingly, multiple emulsions containing HYT showed good antioxidant capacity in the early days of storage, although the level of efficiency depended on the loading method. However, HYT seemed to exert less antioxidant capacity when included in the inner aqueous phase of multiple emulsions.

#### 3.1.2. Gelled Emulsions for HYT

In addition to the expected stability, which is related to the degree of compartmentalization of emulsion-based systems, the bioaccessibility of HYT should also be taken into consideration. In this regard, recent studies showed that gelled emulsions offered a better protection of bioactivity and also a better bioaccessibility compared to simple and double emulsions [96]. While the reducing capacity of the tested emulsions was not altered in the oral phase, in vitro digestion models showed that antioxidant activity was greatly reduced in both the gastric and intestinal phases.

**Figure 2:**
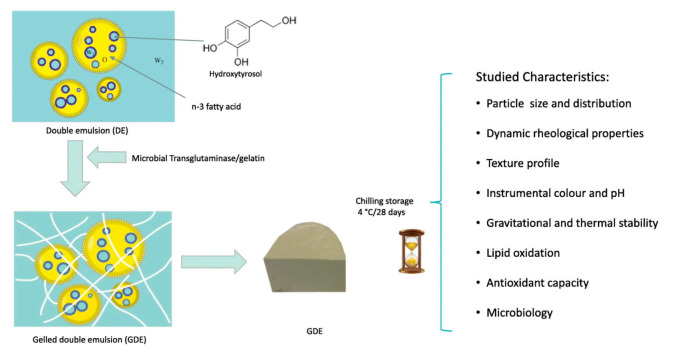
From “Technological characteristics of cold-set gelled double emulsion enriched with n-3 fatty acids: effect of hydroxytyrosol addition and chilling storage” (reproduced with permission from [97]. Copyright 2017, Elsevier).

Moreover, gelled emulsions formulated with perilla oil as the lipid phase, and a source of n-3 fatty acids combined with HYT in the inner aqueous phase stored over 30 days at 4 °C, presented excellent water- and fat-binding properties and, compared to the control without HYT, the encapsulation of the bioactive compound increased its antioxidant capacity by up to 12-fold after preparation, although this declined during the storage period [97] (for a schematization of the system, see Figure 2).

Freire et al. [71] also developed gelled double emulsions as delivery systems for HYT and n-3 fatty acids in healthy pork patties. Specifically, the effect of partial and total replacement of pork back fat with gelled double emulsions, containing perilla oil as the lipid phase and HYT, on the composition and properties of pork patties was investigated. The results demonstrated that the presence of perilla oil as the lipid phase in gelled double emulsions increased the susceptibility to lipid oxidation during the storage of fresh patties. In concert with this, the antioxidant capacity of HYT seemed to be related to its location at the oil–water interphase.

Contextually, Munoz-Gonzales and co-workers [98] formulated emulsion gels with olive oil, polyphenol extracts, and soy protein. Here, the emulsions were assembled by mixing soy protein, alginate, water, and polyphenol extracts, using a homogenizer. Subsequently, the olive oil was gradually added to the mixture to form an emulsion gel. Overall, the phenolic compounds appeared to become trapped in the emulsion gel matrix network and the textural behavior of the emulsion gels may be related to their structural features.

#### 3.1.3. Liposomes for HYT

Among the various positive aspects in the field of delivery systems, liposomes stand out for their advanced ability to deliver active molecules to a site of action. In this respect, research on liposome technology has progressed from “conventional vesicles” to a “new” generation of liposomes, in which long-circulating liposomes are obtained by modulating lipid composition, size, and the charge of the vesicle. Particularly, liposomes with sizes ranging from 100 to 400 nm, with a good encapsulation efficiency of HYT were prepared [104]. Compared to free HYT, HYT-loaded liposomes had better stability and a slower release in vitro. In addition, for these systems, higher radical scavenging activity was found in comparison with free HYT, probably because HYT was fully encapsulated inside liposomes, and lisposomes were able to protect the drug. Thus, prolonging the biological activity of the payload, the obtained preparation had the potential to act as a delivery carrier for further improving the biological functions of HYT in vivo.

HYT was also successfully encapsulated in zwitterionic liposomes [72]. In the cited study, a cytotoxicity assay of the liposomal preparation toward human chondrocytes showed that liposomes were not cytotoxic at the investigated concentrations. In addition to the pure form of HYT, the properties and the antioxidant activity of a series of HYT esters with different carbon chain lengths (C4, C8, C12, and C18) incorporated in liposomal vesicles, were measured [99]. Particularly, the distribution of HYT long chain esters was proven to depend specifically on their lipophilic chain length. Their distribution within the system was found to be very important because it was related to different antioxidant behaviors.

**Figure 3:**
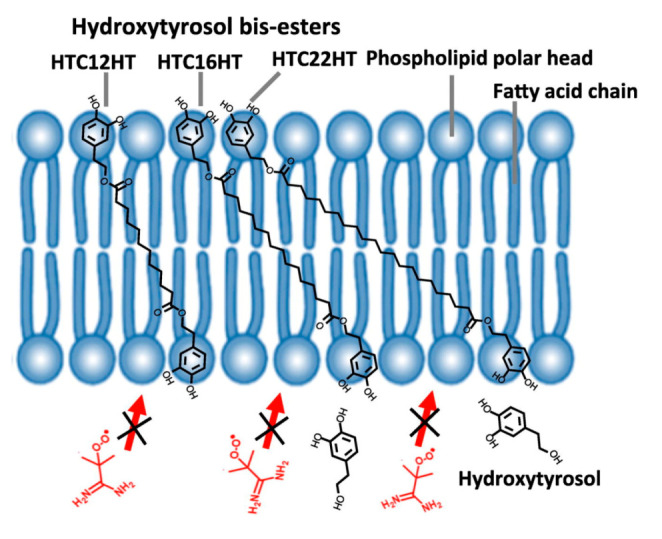
From “A new family of hydroxytyrosol phenolipids for the antioxidant protection of liposomal systems” (reproduced with permission from [103], copyright 2021, Elsevier).

Moreover, HYT esters, synthesized enzymatically from cuphea oil, can be distributed and remain at the aqueous interface of phospholipid–liposomes. This also reduces or prevents liposome rupture by reducing the tension of the liposome membrane, which is an important component for liposome fusion and rupture [102]. Consequently, the investigated HYT esters showed the ability to increase the stability of liposomes. As a consequence, an increased stability of liposomes equates to a longer durability, longer shelf-life, and longer circulation in the system. As matter of fact, HYT esters can be designed for the protection of liposomal systems. Other research has addressed this issue and evaluated the antioxidant activity of formulations against the oxidation induced by 2,2′-azobis(2-amidinopropane) hydrochloride [103] (Figure 3).

The synthetized HYT derivatives were able to protect liposomes from induced oxidation, and the rank of activity was influenced by the alkyl chain length of the HYT esters, the C_12_ derivative being the most active antioxidant, with an increase in the oxidative stability of liposomal preparation of 2.2 times when compared with the control. The incorporation of HYT esters in liposomes improved the antioxidant capacity of the HYT derivatives by about 2.8 times. Thus, HYT-synthesized esters increased general liposomal and oxidative stability and, in parallel, this study also demonstrated the potential to improve the oxidative stability of sensitive fatty acids in food applications. Therefore, by improving the antioxidant activity of hydrophilic phenolic compounds with high free radical scavenging activity (such as HYT), the loaded liposome preparations can effectively be utilized in food industry.

However, the structural changes in the systems have also been linked to the pH of the environment [101]. In fact, both size and surface charge were found to be pH dependent. By taking into account some chemical parameters, it was observed that liposome diameters typically increase from pH 5.5 to pH 10. Zeta potentials values, on the other hand, were found to be well below −25 mV at all explored pH conditions, with the lowest values (and thus, the best liposome stability) at the two extremes of pH 5.5 and pH 10.

Evans et al. [100] explored the bilayer properties of synthesized tyrosol and hydroxytyrosol double-chained phospholipids. In their study, the size, shape, surface charge, and tendency to form supported bilayers on silica surfaces were investigated. The authors performed dynamic light scattering, TEM, zeta potential and fluorescence anisotropy measurements, and concluded that tyrosol and HYT phospholipids formed liposomes of ~85 nm in diameter, with a surface charge of about −25 mV. Furthermore, it was found that the surface adsorption of both liposomes was influenced by the phospholipid concentration and the presence of calcium.

The same research team enzymatically trans-esterified both HYT and tyrosol from olive oil processing into effective lipophilic antioxidants using cuphea oil [102]. Their work demonstrated that HYT and tyrosol esters partitioned and resided at the aqueous interface of phospholipid liposomes. The partitioning was shown to be similar for both tyrosol and HYT derivatives. Overall, both tyrosol and HYT derivatives showed the ability to reduce or prevent the rupture of liposomes. Considering that tyrosol and HYT esters synthesized from cuphea oil increased liposomal stability, this suggests that this type of liposome can be potentially used in the food industry.

#### 3.1.4. Solid Particles for HYT

Solid particles represent also good delivery systems. For example, the encapsulation of HYT in pectinate beads was studied and outcomes showed a good entrapment efficiency and in vitro release [106]. In fact, beads were able to entrap HYT in sufficient amounts to reach the colon (around 50%), maintaining concentrations of pectin-bound HYT after two hours at gastric pH. Thus, considering that, thanks to HYT-loaded pectinate gel beads, a good amount of HYT was able to reach the colon (20–30%), this system demonstrated to be a good colon-targeted delivery system for HYT in order to help prevent or relieve chronic inflammatory bowel disease.

Flaxseed protein isolate and its phenolic complexes with HYT were studied with the aim of developing plant-based natural emulsifiers with improved interfacial and oxidative activity [110]. Particularly, the complexation of flaxseed protein isolate with HYT significantly increased the diffusion rate at a low emulsifier concentration (0.1 mg mL^−1^). Moreover, the emulsions stabilized by the formation of the complexes between flaxseed protein isolate and HYT had a higher antioxidative stability compared to the control, but, on the contrary, the stability of the system resulted in being lower. Therefore, it can be considered true that the fabrication of specific complexes can allow HYT delivery, but, at the same time, both the advantages and limitations (as in this case) of the final product must be taken into account, where formulation definition represents the most important step for effective delivery systems.

**Figure 4:**
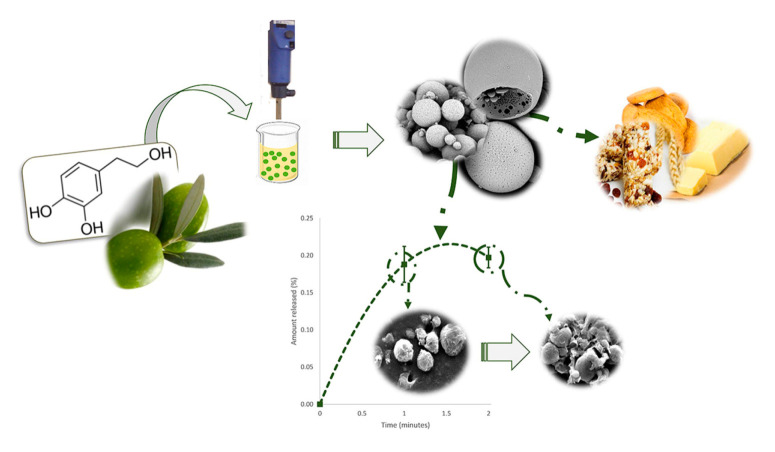
From “Inclusion of hydroxytyrosol in ethyl cellulose microparticles: In vitro release studies under digestion conditions” (reproduced with permission from [109], copyright 2018, Elsevier).

Ethyl cellulose is another suitable material for the production of solid particles. In this context, microparticles were produced using the double emulsion solvent evaporation technique of Paulo and Santos [109] (see Figure 4).

From the investigation it was ascertained that the maximum encapsulation efficiency and product yield were 88% and 82%, respectively. The HYT-loaded ethylcellulose microparticles were almost stable in the temperature range of 30 °C to 280 °C, and the antioxidant capacity remained above 50%, which is within the effective concentration range for many beneficial biological effects. Moreover, in the study, gastrointestinal resistance was achieved as well. HYT was demonstrated to be able to form a 1:1 inclusion complex with β-cyclodextrin, thus leading to a freeze-drying and spray-drying solid particle system for its delivery, while retaining its antioxidant capacity [108].

Moreover, it is also possible to co-deliver HYT with other payloads, such as syringopicroside [107]. Another nanocarrier for the delivery of HYT is characterized by the presence of monomethoxypolyethylene glycol-poly (lactic-co-glycolic acid) (mPEG-PLGA). The obtained system resulted in nanoparticles with an encapsulation efficiency of 32% for both HYT and syringopicroside; in contrast, drug loading was 12%. Moreover, in vitro drug release patterns showed relatively prolonged drug release, while pharmacokinetic studies demonstrated that the half-life and residence time of the co-loaded nanoparticles, in vivo, were prolonged significantly. For this reason, this delivery system demonstrated the potential to improve the bioavailability of two bioactives, showing a sustained release effect, prolonging drug circulation time in blood. In addition to conventional precipitation methods for loaded solid particles, there is an alternative approach for solid lipid particles, namely membrane emulsification [105]. This method ensures good encapsulation efficiency (< 90%, when the process is carried out at room temperature), low energy consumption (1.1·10^6^ J m^−3^), and high dispersed phase concentration (> 40%). In addition, it is not complicated and is therefore extremely attractive for the production of lipid matrix-based carriers.

### 3.2. Hydrophobic Phenols: The Case of Curcumin

Having established CUR’s safety in normal doses [111], it is possible to affirm that this bioactive molecule represents a useful tool as a dietary supplement. Apart from following re-called formulations, as in the case of HYT, many curcumin-based supplements are widely available on the market and many examples can also be easily found on the web, such as Tumeric Complex (Bioeva, Rome, Italy), Curcumin 95 (Jarrow Formulas, Los Angeles, CA, USA), and Curcuma Forte (LongLife, Milan, Italy) [112]. In addition, in this case, starting from the well-studied available delivery systems, scientific innovations for CUR supplementation represent a very interesting research field. The main colloid-based delivery systems discussed for CUR are summed up in Table 2.

#### 3.2.1. Macroemulsions for CUR

As expected, among the suitable solutions, emulsion-based techniques represent a useful approach for the realization of CUR delivery systems. In particular, *O*/*W* macroemulsions provided protection to the encapsulated CUR against chemical degradation [118]. This system was quite simple to fabricate, which would be advantageous for many commercial applications with the aim of enhancing the bioaccessibility of the molecule [119]. To ensure a high transfer of CUR from the initial pure powder formulation into emulsion lipid droplets, a suitable temperature and droplet size needs to be found [121]. CUR’s solubility in emulsion was found to increase with temperature and with the type of emulsifier, and was found to be higher with surfactants than with proteins. Bioaccessibility, on the other hand, increased using proteins as emulsifiers since they protected CUR from chemical degradation in simulated gastrointestinal conditions [120].

Feng et al. [114] developed CUR-rich emulsions using debranched starch as an interface material. These emulsions were more stable than those stabilized with Tween 80 and lectin. Interestingly, molecular dynamics simulations showed that the water bridge between the debranched starch and CUR may play an important role in the complexation process, thus contributing to better performance of the emulsion.

In another study, Kharat and co-workers [116] considered the effect of droplet surface area on the kinetics of CUR degradation using emulsions with different mean droplet diameters. The authors observed that the rate of CUR degradation increased with decreasing droplet size, and they concluded that this was attributable to the fact that CUR exchange between the interior and exterior was faster in small droplets.

Li et al. [117] investigated the effect of the degree of substitution of octenyl succinic anhydride (OSA) on the properties of starch microparticles (SMPs)-stabilized emulsions encapsulating CUR. Overall, they found that higher degrees of substitution led to a lesser extent of OSA-modified SMPs digestion, less droplet flocculation, and coalescence during the digestion and the higher bioaccessibility of CUR in the emulsion.

In this context, Cheng et al. [113] developed tunable high internal phase emulsions (HIPEs) formulated using lactoferrin-gum arabic complexes, where CUR was used as the model lipophilic nutraceutical to investigate the potential of HIPEs to improve the photo-stability of encapsulated bioactives. The authors found that HIPE-stabilized complexes showed gel-like structures, good creaming stability, and excellent environmental stability, showing potential use in semi-solid foods to improve textural characteristics or nutritional profiles. Lastly, Hamad et al. [115] proposed a novel approach to prepare spray-dried encapsulated CUR powders. Specifically, the effects of surfactants (Tween 80, lecithin, and chitosan) on the characteristics of CUR-based emulsions, as well as on the physicochemical properties of the resulting spray-dried encapsulated powder, were determined. Overall, the results of this study can be considered a guide for the development of a stable formulation of CUR emulsions that can be converted into an encapsulated powder form using spray drying.

#### 3.2.2. Nanoemulsions for CUR

When encapsulated in nanoemulsions, the dispersibility of CUR in water can be increased 1400-fold compared to the raw biomolecule [128]. Sari et al. reported that CUR is slowly released from nanoemulsions in simulated gastro-intestinal digestion conditions [43]. Accordingly, nanoencapsulation of highly lipophilic and unstable compounds, such as CUR, is an effective approach to increase the solubility, bioaccessibility, and to protect the molecule from degradation. The choice of optimal formulation represents an essential aspect for the stability of CUR-loaded nanoemulsions and to preserve the antioxidant capacity of loaded CUR [122]. In particular, Tween 20 and sucrose monopalmitate allow to obtain delivery systems with a high encapsulation efficiency. The addition of lecithin ensures the formation of long-term stable nanoemulsions that can also preserve the antioxidant capacity of the bioactive compound.

Kharat et al. [125] prepared CUR nanoemulsions with added antioxidants and found that CUR retention during storage declined differently using different antioxidants. The stabilizing effect of ascorbic acid increased with concentration (0–300 μM). Taken together, these observations form the basis for the production of CUR-enriched foods and beverages with increased bioactivity.

**Figure 5:**
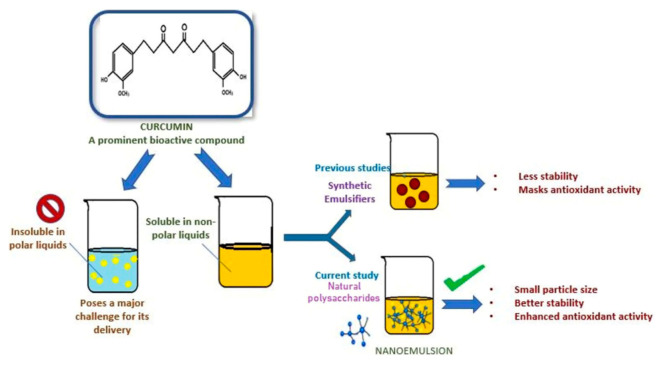
From **“**Exploration of polysaccharide based nanoemulsions for stabilization and entrapment of curcumin” (reproduced with permission from [129], copyright 2020, Elsevier).

Other authors enzymatically prepared monoacylglycerides and diacylglycerides structured with conjugated linoleic acid (CLA), medium chain fatty acids (MCFA), and ω-3 fatty acids (ω-3 FA), used to assemble nanoemulsions employed as carrier systems of lipophilic active compounds with a low bioavailability, such as CUR, which need to be protected from the environment [124]. Contextually, Richa et al. [129] developed polysaccharide-based nanoemulsions for the stabilization and entrapment of CUR (see Figure 5).

In that work, the authors investigated two different oil carriers, namely olive and castor oil, and various polysaccharides, namely levan, fucoidan, alginate, guar gum, and κ-carrageenan, for their entrapment efficiency for CUR. Interestingly, the smallest particle size was observed after ultrasonic treatment when fucoidan was used as an emulsifier. It was also found that the highest encapsulation efficiency was exhibited by κ-carrageenan irrespective of the carrier oil. Additionally, the release kinetics of the nanoemulsions were in direct correlation with the encapsulation efficiency. Of note, a synergistic increase in the antioxidant potential of the polysaccharide-based nanoemulsions containing CUR was detected. In another study, Espinosa-Andrews et al. [126] aimed to determine the optimal conditions to produce CUR nanoemulsions using ultrasonication stabilized with hydroxylated lecithin, using a response surface methodology to evaluate some physical characteristics. The overall results highlighted that the nanoemulsions remained stable during 15 days of storage at 20 °C and against aggregation in a pH range from 7.0 to 3.0. The authors concluded that this type of nanoemulsion system could be used as a natural colorant in beverages.

The potential of heterogeneous systems, such as oil-in-water (*O*/*W*) nanoemulsions, has been exploited as an oral delivery system for CUR. Among the proteins, sodium caseinate is generally used as food ingredient in food industry. The combination of nanoemulsions, stabilized by a blend of caseinate and Tween 20, demonstrated that the presence of Tween 20 ensured steric stabilization, promoting the use of sodium caseinate as an emulsion stabilizer [11]. In a recent study, Cuomo et al. [123] demonstrated that the blend of protein/surfactant-stabilized nanoemulsions is a suitable solution to deliver CUR. The nanoemulsions proposed in the study provided a high intake of CUR with a low fat content. The use of such delivery systems helps to overcome the limitations of oral bioavailability associated with the low solubility of some compounds in foods and beverages. The results of the study showed that nanoemulsions stabilized with proteins and surfactants are a suitable solution for CUR. The use of such delivery systems helps to overcome the limits in oral bioavailability related with the scarce solubility of some compounds in food preparations and beverages.

#### 3.2.3. Pickering Emulsions (PE) for CUR

CUR can also be successfully encapsulated in more complex emulsion-based systems, such as in PE. In this respect, emulsions stabilized with chitosan/tripolyphosphate nanoparticles showed a positive effect on the emulsion stability against aggregation under different conditions [41], and the release profile of CUR from this system showed a sustained release over an extended period of time.

Another effective stabilizer material is starch [133]. Starch-stabilized PE showed an encapsulation efficiency of CUR of about 80%, retaining and gradually releasing the bioactive during storage and simulated oral (from 69.6% to 95.3%) and gastric digestion (from 82.4% to 86.2%), reaching the intestine, which represents a desirable trait since most nutrient adsorption occurs there.

Milled cellulose-stabilized PE are a novel food-grade formulation for the encapsulation and delivery of CUR, as reported in a study by Lu and Huang [131]. The study showed that the bioaccessibility of CUR encapsulated in these systems was higher than in conventional emulsions. The use of nanocellulose-stabilized PE has also been proposed for CUR encapsulation [127], showing positive effects on storage stability at different pH values, on sustained release, and for in vitro cytotoxicity against fungi and pathogen bacteria (Gram-positive and Gram-negative), demonstrating antifungal and antimicrobial effects

Wei and Huang [134] developed high internal phase PE stabilized by ovotransferrin–gum arabic particles as CUR delivery vehicles. This PE significantly improved both the extent of lipolysis and CUR bioaccessibility. In the study by Lv and co-workers [132], whey protein isolate (WPI) gel particles were fabricated via high hydrostatic pressure treatment and homogenization and the potential of using the particles as food-grade stabilizers to form PE and emulsion gels were studied, also in relation to CUR load.

In a parallel study, chitosan/gum arabic nanoparticles were prepared and their potential use in stabilizing PE and the delivery of CUR were evaluated [130]. It was found that chitosan and gum arabic mainly interacted electrostatically, and the obtained nanoparticles were about 100 nm. This type of PE exhibited high CUR loading and enhanced CUR protection during storage and release during in vitro digestion.

**Figure 6:**
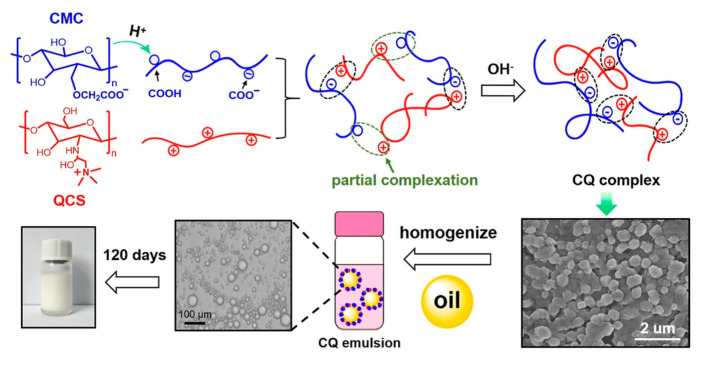
From “Tuning complexation of carboxymethyl cellulose / cationic chitosan to stabilize Pickering emulsion for curcumin encapsulation” (reproduced with permission from [135], copyright 2021, Elsevier).

Recently, Zhu et al. [135] tuned the complexation of carboxymethyl cellulose/cationic chitosan to stabilize a PE suitable for CUR encapsulation (see Figure 6).

These emulsions exhibited gel-like behavior and were stable to various environmental influences, such as pH, salt, temperature, and long-term storage. In addition, the authors showed that they efficiently encapsulated CUR, which had a lower rate of degradation.

#### 3.2.4. Multilayer Emulsions for CUR

Layer-by-layer assembly can successfully change the surface characteristics of CUR-loaded nanoemulsions, allowing the formation of chitosan- and alginate-based multilayer emulsions [139]. These nanosystems were found to be very stable to changes in temperature and pH under storage conditions. In particular, the deposition of chitosan and alginate leads to the formation of a stable multilayer structure, mainly due to electrostatic interactions between the polyelectrolytes.

Lower bioaccessibility values were observed in in vitro digestion for multilayer nanoemulsions when compared to simple nanoemulsions, [140] due to the fact that polyelectrolyte layers prevented the release of CUR from the multilayer. The degradation of CUR during digestion was also lowered by the protective effect of the multilayer, which sterically hindered CUR from enzymatic or free radical action. Whey proteins isolate-stabilized chitosan-based multilayer nanoemulsions can also significantly increase CUR antioxidant activity, enhancing also its apparent permeability coefficient in Caco-2 cells by 1.55-fold, thus improving its anticancer activity [138].

In a study by Leiva-Vega et al. [136], the authors investigated the influence of interfacial structure on physical stability and the antioxidant activity of CUR multilayer emulsions. Specifically, CUR was dissolved in coconut oil and encapsulated in multilayer emulsions, prepared via layer-by-layer deposition. The antioxidant activity of the emulsified CUR was maintained during a 6-day light exposure, where it decreased significantly in free CUR from day 0 onwards.

**Figure 7:**
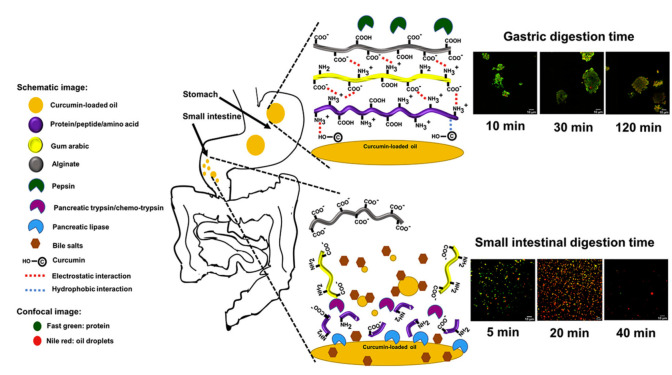
From “An efficient small intestine-targeted curcumin delivery system based on the positive-negative-negative colloid interactions” (reproduced with permission from [137], copyright 2021, Elsevier).

Sabet et al. [137] developed a positive–negative–negative delivery system (as shown in Figure 7) to protect CUR against the gastric phase with the aim of releasing it in the small intestine.

The results showed that this system was more resistant against gastric proteolysis compared with a primary emulsion and two bilayer emulsions. Remarkably, almost 84% of the original CUR was efficiently released in the small intestine, compared to less than 20% in other delivery systems.

#### 3.2.5. Liposomes for CUR

In recent research, coated vesicles were demonstrated to be a more efficient delivery systems than conventional ones [142]. In the study, it was shown that CUR, loaded in the bilayer of liposomes coated with chitosan, was better absorbed through the digestive tract than CUR loaded into bare anionic liposomes, due to interactions occurring among chitosan and the other charged molecules of the digestion fluids.

Li et al. [144] used thiol derivatives of chitosan to cover liposomes loaded with CUR and the liposomes resulted effective for the treatment of MCF-7 cell line. Tai and colleagues [145] demonstrated that molecular weight and the concentration of chitosan play an important role in stabilizing this kind of system. In particular, both low- and high-molecular weight coatings resulted in better photo and thermal stability.

In recent work, a long-circulating delivery system of liposomal CUR by coating with albumin was developed [146]. In this work, the authors found that liposomes coated with albumin were more spherical, more homogeneous in size, and significantly larger than uncoated counterparts. In addition, the authors investigated the impact of the coating albumin on the release of CUR and phagocytosis using mouse Raw264.7 macrophages in vitro. The overall results demonstrated that the presence of albumin enhanced liposome structure stability and slowed down the release of CUR, and that macrophage phagocytosis of CUR-loaded liposomes was significantly reduced.

In another study, folated pluronic (FA-F127)-modified liposomes for the delivery of CUR were fabricated [39]. It was demonstrated that the effects of FA-F127 modifications on average particle size, PDI, CUR encapsulation efficiency, and microstructure were not significant. Furthermore, compared with nonfolated F127 liposomes, FA-F127 liposomes exhibited significantly higher cytotoxicity towards KB cancer cells.

Contextually, De Leo and co-workers [143] loaded CUR in Eudragit-coated liposomes to create a gastro-resistant carrier, able to protect its load from degradation and free it at the site of absorption in the colon region. The authors investigated the physicochemical properties of the systems and studied the uptake of vesicles by Caco-2 cells and the antioxidant activity in cells. The results showed that the polymeric coating dissolved at pH > 7.0, releasing liposomes and allowing them to enter Caco-2 cells.

Chen et al. [42] investigated the stability and the release of CUR-loaded liposomes with varying contents of hydrogenated phospholipids, demonstrating that the latter increased liposomal CUR encapsulation and improved the stability of liposomes. In addition, hydrogenated phospholipids slowed down CUR release from liposomes in simulated digestion. Moreover, the modified phospholipids, contributed to denser lipid packing in liposomal membranes. In another paper, the authors modified CUR-loaded liposomes with edible compounds to improve their ability to cross the blood–brain barrier [141]. More in detail, the research explored the potential of food grade sialic acid, polymerized sialic acid, and their oxidized forms to conjugate with wheat germ agglutinin on liposomes to deliver CUR to the brain. The results showed that CUR was entrapped in liposome with high encapsulation efficiency. These liposomes showed a good permeation rate with respect to the endothelial cells of the blood–brain barrier. Overall, this study highlighted how the surface modifications can improve the function of liposomes as carriers for brain disease.

#### 3.2.6. Solid Particles for CUR

Compared to the emulsion-based methods, nanoprecipitation approach is simple, cost-effective and does not require energy inputs. By selecting appropriate solvent mixtures, it is possible to first obtain the precipitation of uniform drug-loading nanoparticles and then of the polymer molecules covering the particles, thus forming drug–core polymer–shell structures.

Using this method, extremely stable and high CUR loading nanoparticles with a high encapsulation efficiency were obtained [151].

In particular, considering that the shellac natural resin, used in both the pharmaceutical and food industries, is more soluble in weak alkaline conditions, by using PBS or HEPES buffers at pH 7.4 it was demonstrated that CUR precipitated first, followed by the polymer [45]. Solid lipid nanoparticles prepared using tristearin and polyethylene glycole-enriched emulsifiers have been demonstrated to be able to control the absorption of orally administered CUR under simulated gastrointestinal conditions, enhancing its bioavailability [148].

Other studies showed that encapsulation in modified rice starch was able to improve CUR’s solubility, stability, bioavailability, and biocompatibility [152]. The obtained nanoparticles possessed excellent colloid stability, which protected the bioactive substance from UV degradation and heat exposure. Thus, CUR loaded in nanoparticles showed better antioxidant activity than free CUR.

In particular, a recent study confirmed that the encapsulation approach based on more complex matrices could enhance the effectiveness of the final system [154]. As a matter of fact, the zein–shellac binary matrix employed for CUR’s encapsulation provided a higher encapsulation efficiency than that of individual components. The system prevented also CUR degradation, induced by thermal treatment and UV light radiation, and exhibited a great ability to sustain the release of the bioactive in both PBS medium and simulated gastrointestinal tract conditions.

Through a simple pH-shift method, CUR-loaded nanoparticles stabilized using sodium caseinate and gum arabic were prepared [153]. These systems were stable in a pH range of 2–7 and in presence of NaCl. Moreover, they also showed high encapsulation efficiency. Similarly, Zhan et al. [157] entrapped CUR in whey protein isolate (WPI) and zein composite nanoparticles using a pH-driven method. The results highlighted that nanoparticles exhibited the smallest size at a WPI-to-zein mass ratio of 8:2. Furthermore, the increase in the zein level improved the thermal stability of CUR-loaded WPI–zein composite nanoparticles. In addition, it was found that the solubility of CUR was significantly enhanced by its encapsulation in WPI–zein composite nanoparticles.

**Figure 8:**
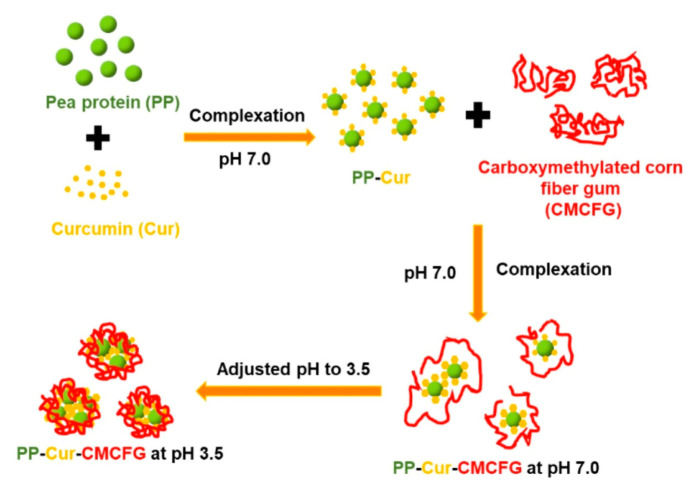
From “Core-shell pea protein-carboxymethylated corn fiber gum composite nanoparticles as delivery vehicles for curcumin” (reproduced with permission from [155], copyright 2020, Elsevier).

In the work by Yuan et al. [156], the authors fabricated and characterized zein nanoparticles with a dextran sulfate coating as vehicles for the delivery of CUR. Specifically, zein/dextran sulfate composite nanoparticles were fabricated via an antisolvent precipitation method at pH 4.0 with an optimal zein-to-dextran sulfate ratio of 1:2 (*w*/*w*). These nanoparticles exhibited good stability relative to pH, heating, and storage. Interestingly, the nanoparticles displayed no toxicity toward normal colonic epithelial cell lines and behaved as efficient vehicle for CUR, with high encapsulation efficiency. In this context, Wei et al. [155] developed core–shell pea protein–carboxymethylated corn fiber gum composite nanoparticles as a carrier for CUR (see Figure 8).

In this work, pea protein–CUR complexes were formed at pH 7.0 and successively coated with carboxymethylated corn fiber gum via hydrophobic interactions to form complexes. These nanocomposites exhibited excellent encapsulation performance for CUR, showing also good water dispersibility and high chemical/thermal stability. Furthermore, these nanocomposites showed even higher antioxidant and radical scavenging activities than simple CUR. In another work, the authors proposed solid lipid nanoparticles stabilized by sodium caseinate–lactose Maillard conjugate encapsulating CUR. In this study, it was found that the entrapment efficiency was more than 90% when CUR was 2.5% and 5.0% of the lipid mass. In addition, these nanoparticles were stable during 30-day storage and also greatly enhanced the antioxidant activity of encapsulated CUR.

## 4. Concluding Remarks

This review provides an overview of the latest results on available colloid carrier systems for bioactive substances and presents two representative studies on hydrophilic (hydroxytyrosol) and lipophilic (curcumin) molecules. Considering the beneficial effects of their consumption, improving the bioavailability and efficacy of in vivo bioactives through appropriate delivery systems is a very interesting challenge in both the food and pharmaceutical fields. In this sense, colloids have offered several alternatives for the realization of a wide range of delivery systems in recent years. In particular, these substances can be used for the preparation of colloid systems, such as molecular complexes, emulsion-based systems, particles, and vesicles, and all are suitable solutions to achieve the above-mentioned goal. It is, therefore, clear that all reported delivery systems show promising and attractive benefits for the protection and release of both hydrophilic and lipophilic bioactive substances. This is a very important aspect in the context of promoting novel healthy food supplements. However, this research is in constant development and this is not the end point. Instead, it could be a starting point for researchers to improve and/or develop novel colloid-mediated delivery systems.

## Figures and Tables

**Table 1:** Main colloid-mediated delivery systems for hydroxytyrosol.

Delivery System	Applications	References
Macroemulsions	Antioxidant activity, releasing efficacy, shelf life, solubility and gastrointestinal stability.	[91,92,93,94,95]
Multiple emulsions	Antioxidant activity, loading losses	[93,94]
Gelled emulsions	Antioxidant activity, HYT losses and HYT release	[71,96,97,98]
Liposomes	Stability, antioxidant activity	[72,99,100,101,102,103,104]
Solid particles	Loading capacity, in vitro antioxidant activity	[105,106,107,108,109,110]

**Table 2:** Main colloid-mediated delivery systems for curcumin.

Delivery System	Applications	References
Macroemulsions	Encapsulation efficiency, stability, and bioaccessibility	[113,114,115,116,117,118,119,120,121]
Nanoemulsions	Dispersibility, digestion, release kinetic, stability, antioxidant capacity, encapsulation efficiency	[43,122,123,124,125,126,127,128,129]
Pickering emulsions	Stability, release kinetic, encapsulation efficiency, temperature response, digestibility, bioaccessibility, cytotoxicity, anticancer/antifungal/antimicrobial activities	[41,127,130,131,132,133,134,135]
Multilayer emulsions	Stability, bioaccessibility, antioxidant activity, digestibility, permeability, and bioeffectives	[136,137,138,139,140]
Liposomes	Solubility, stability, anticancer activity, adsorption, release kinetic	[42,141,142,143,144,145,146,147]
Solid particles	Encapsulation efficiency, bioaccessibility, stability, antioxidant activity, biocompatibility, and in-vitro gastrointestinal release kinetic	[45,148,149,150,151,152,153,154,155,156,157]
